# Pan-Cancer analysis and experimental validation identify the oncogenic nature of ESPL1: Potential therapeutic target in colorectal cancer

**DOI:** 10.3389/fimmu.2023.1138077

**Published:** 2023-03-16

**Authors:** Yuchen Zhong, Chaojing Zheng, Weiyuan Zhang, Hongyu Wu, Meng Wang, Qian Zhang, Haiyang Feng, Guiyu Wang

**Affiliations:** ^1^ Cancer Center/Department of Colorectal Cancer Surgery, The Second Affiliated Hospital of Harbin Medical University, Harbin, Heilongjiang, China; ^2^ Department of Colorectal Cancer Surgery, The Cancer Hospital of University of Chinese Academy of Sciences (Zhejiang Cancer Hospital), Institute of Basic Medicine and Cancer (IBMC), Chinese Academy of Science, Hangzhou, Zhejiang, China

**Keywords:** cell cycle, ESPL1, pan-cancer, patient derived organoids, cancer therapy

## Abstract

**Introduction:**

Extra spindle pole bodies like 1 (ESPL1) are required to continue the cell cycle, and its primary role is to initiate the final segregation of sister chromatids. Although prior research has revealed a link between ESPL1 and the development of cancer, no systematic pan-cancer analysis has been conducted. Combining multi-omics data with bioinformatics, we have thoroughly described the function of ESPL1 in cancer. In addition, we examined the impact of ESPL1 on the proliferation of numerous cancer cell lines. In addition, the connection between ESPL1 and medication sensitivity was verified using organoids obtained from colorectal cancer patients. All these results confirm the oncogene nature of ESPL1.

**Methods:**

Herein, we downloaded raw data from numerous publicly available databases and then applied R software and online tools to explore the association of ESPL1 expression with prognosis, survival, tumor microenvironment, tumor heterogeneity, and mutational profiles. To validate the oncogene nature of ESPL1, we have performed a knockdown of the target gene in various cancer cell lines to verify the effect of ESPL1 on proliferation and migration. In addition, patients’ derived organoids were used to verify drug sensitivity.

**Results:**

The study found that ESPL1 expression was markedly upregulated in tumorous tissues compared to normal tissues, and high expression of ESPL1 was significantly associated with poor prognosis in a range of cancers. Furthermore, the study revealed that tumors with high ESPL1 expression tended to be more heterogeneous based on various tumor heterogeneity indicators. Enrichment analysis showed that ESPL1 is involved in mediating multiple cancer-related pathways. Notably, the study found that interference with ESPL1 expression significantly inhibited the proliferation of tumor cells. Additionally, the higher the expression of ESPL1 in organoids, the greater the sensitivity to PHA-793887, PAC-1, and AZD7762.

**Discussion:**

Taken together, our study provides evidence that ESPL1 may implicate tumorigenesis and disease progression across multiple cancer types, highlighting its potential utility as both a prognostic indicator and therapeutic target.

## 1 Introduction

It is well known that cancer incidence is significantly associated with age. To date, cancer remains the second leading cause of human death (1). Despite the advances in medical technology and the increasing number of cancer treatment options, a large number of patients are still diagnosed at an advanced stage when treatment approaches are often not feasible, eventually resulting in cancer-related death. Given that surgery and chemotherapy alone are not enough to save cancer patients, there is a need to develop more cancer treatment options. Therefore, this calls for studies to explore the mechanisms of cancer development at the molecular level for effective diagnosis and treatment.

ESPL1 (extra spindle pole bodies like 1) is a protein-coding gene whose related pathways are mitotic G1-G1/S phases and cell cycle. Notably, ESPL1 is regulated by at least two independent mechanisms. First, it is inactivated *via* interaction with securin/PTTG1, which probably covers its active site (2). It should be noted that its association with PTTG1 is not only inhibitory since PTTG1 is also required for ESPL1 activation, and thus the enzyme is inactive in cells in which PTTG1 is absent. Therefore, degradation of PTTG1 at anaphase liberates ESPL1 and triggers RAD21 cleavage. Second, phosphorylation at Ser-1126 inactivates it. The complete phosphorylation during mitosis is removed when cells undergo anaphase. Studies have proposed that activating the enzyme at the metaphase-anaphase transition requires the removal of both securin and inhibitory phosphate (3–5). A previous cancer study discovered frequent alterations in STAG2 and ESPL1 in bladder cancer, which suggests that it may be involved in bladder tumorigenesis through sister chromatid cohesion and segregation process (6). In addition, two other previous studies concluded that ESPL1 might be a prognostic biomarker in malignant glioma and endometrial cancer (7, 8).

Considering that ESPL1 is still inadequately studied in cancer and there are no relevant pan-cancer analyses, the main aim of this study was to perform a systematic full-scale pan-cancer analysis of tumor samples from public databases. Specifically, we explored the expression and prognostic significance of ESPL1 in various human malignancies using data from The Cancer Genome Atlas (TCGA). Furthermore, we evaluated the association of ESPL1 expression with tumor-infiltrating immune cells and immune-related genes, and then explored the association between ESPL1 expression and tumor mutational load (TMB), microsatellite instability (MSI), mutant-allele tumor heterogeneity (MATH), and homologous recombination deficiency (HRD). Moreover, we identified ESPL1 specific genes and signaling pathways that regulate cancer progression and finally performed a drug correlation analysis. Collectively, the findings of this study reveal that ESPL1 is associated with tumorigenesis and progression in a variety of cancers, which suggests that it is a potential prognostic marker.

## 2 Materials and methods

### 2.1 Data collection and processing

Standardized pan-cancer dataset was downloaded from the Xena functional genomics explorer (https://xenabrowser.net/) database, followed by extraction of the expression data of ENSG00000135476 (ESPL1) gene in each sample. Next, log2(x+1) transformations were performed for each expression value. Notably, the expression data of 33 cancer species were obtained. Due to the small sample size of normal tissues from the TCGA database, we further retrieved normal tissue expression data from the GTEX database (9). The abbreviations for the names of the cancers are in **Supplementary Table 1**. For colorectal cancer, liver cancer, lung cancer, and cervical cancer, we also compared gene expression levels using GEO data. These eight GEO datasets are GSE39001 and GSE6791 for cervical cancer (10, 11), GSE112790 and GSE45267 for liver cancer (12, 13), GSE68571 and GSE75037 for lung cancer (14, 15), and GSE24550 and GSE21815 for colorectal cancer (16, 17). To ensure data comparability, we performed normalization of the data using the “preprocessCore” package. For batch effects, we utilized the “removeBatchEffect” function from the “limma” package for removal.

### 2.2 Gene expression and clinical and survival analysis

The tumor cell line expression matrix was obtained from the CCLE dataset (https://portals.broadinstitute.org/ccle/about), and analysis was conducted using “ggplot2” R package (v3.3.3) (18, 19).

Next, we obtained a high-quality prognostic dataset from the TCGA prognostic study previously reported by Liu J et al (20). The Cox proportional hazards regression model was then built using the “coxph” function of the “survival” R package (version 3.2-7) to analyze the relationship between gene expression and prognosis in each tumor. The function “surv_cutpoint” calculates the optimal cut point for survival analysis and restricts the group proportion such that a subgroup cannot exceed 60% of the total sample size.

Univariate Cox regression analysis and forest plots generated through the “forestplot” R package were used to display the P value, HR, and 95% CI of each variable. For the multivariate analysis, we utilized the R package “coxph” for data processing and incorporated various factors such as TNM staging, clinical staging, tumor grade, tumor location, pathological type, age, and sex for different cancer types. Finally, the “survminer” package was used to visualize the results of the multivariate analysis.

For receiver operating characteristic (ROC) analysis, we performed the analysis using the “timeROC” package (version 0.4) in R language and generated the graphs using the “pdf” and “plot” functions. The ROC was constructed based on three primary parameters: survival status, survival time, and ESPL1 expression level. The training and testing sets were randomly partitioned using the “caret” package, with a ratio of 70:30 for the partitioning. Specifically, the “createDataPartition” function was employed for random partitioning, with the survival outcome as the sampling parameter. 70% of the samples were designated as the training set and the remaining 30% as the testing set.

For age comparison, we divided the samples into high and low expression groups based on the median ESPL1 expression level and compared the age distribution between the two groups. For comparison between genders, we directly compared the ESPL1 expression levels between males and females in each cancer type.

### 2.3 Genetic heterogeneity analysis

Homologous recombination deficiency (HRD) data for each tumor was obtained from previous studies (21). We then integrated the HRD and gene expression data of the samples, and then log2(x+1) was further used to transform each expression value.

MuTect2 software processed the level 4 simple nucleotide variation dataset downloaded from TCGA, calculated the tumor mutation burden (TMB) and mutant-allele tumor heterogeneity (MATH) for each tumor using the TMB and inferHeterogeneity function of the R package maftools (version 2.8.05), and combined the TMB and MATH score with gene expression data (22). A log2(x+1) transformation was further applied to each expression value.

The microsatellite instability (MSI) scores for each tumor were obtained from previous studies and integrated with the available data, and finally log2(x+1) transformations were performed (23).

### 2.4 Immune analysis

The expression data of two types of immune checkpoint pathway genes [inhibitory (24) and stimulatory (25)] and five types of immune pathway genes [chemokine (26), receptor (18), MHC (21), immuno-inhibitor (24), and immuno-stimulator (27)] in each sample were extracted from the downloaded TCGA dataset, and all normal samples were filtered. Log2(x+1) transformation was performed on each expression value, and the Pearson correlation between ENSG00000135476 (ESPL1) and marker genes was calculated. Next, the deconvo_xCell method of the R package IOBR (version 0.99.9) was used to analyze the relationship between immune cells and the expression of ESPL1 (24, 28). We used the false discovery rate (FDR) method to correct the p-values when performing the correlation analysis to ensure statistical accuracy. In more detail, the ‘corr.test’ function in the R package ‘psych’ is used for correlation analysis, with the ‘adjust’ parameter set to ‘fdr’.

Notably, the ESTIMATE algorithm includes three scores: immune score (assessment of immune cell infiltration level); stromal score (assessment of immunity of stromal components); and ESTIMATE score. The “Estimate” R package evaluates the above three scores for each TCGA sample (29).

### 2.5 Protein–protein interaction analysis

The protein-protein interaction (PPI) network was established using the Search Tool for the Retrieval of Interacting Genes (STRING) (https://cn.string-db.org/) with the following input parameters: “evidence”, “experiments”, and “low confidence level”. A total of 31 nodes were finally obtained and subjected to enrichment analysis. The Kyoto Encyclopedia of Genes and Genomes (KEGG) results were replotted by http://www.bioinformatics.com.cn, a free online platform for data analysis and visualization.

### 2.6 Enrichment analyses and similar genes

The GEPIA2 (http://gepia2.cancer-pku.cn/#index) database was used to obtain the top 200 genes similar to ESPL1 based on the TCGA dataset using the “Similar Gene” function (30). The heat map of similar genes and ESPL1 correlation was also obtained using the “Gene_Corr” function of TIMER2.0 database (http://timer.cistrome.org/) (31–33). The ESPL1 negatively correlated genes were identified using the “psych” package in R.

Next, Webgestalt (http://www.webgestalt.org/) and “clusterprofile” package in R were used for enrichment analysis of the 200 similar genes (34, 35). The basic parameters were Homo sapiens, ORA, and pathway-KEGG, whereas the reference set was genome encoding-protein. In addition, the advanced parameters were set to FDR < 0.05.

### 2.7 Drug sensitivity analysis

The Genomics of Drug Sensitivity in Cancer (GDSC) (https://www.cancerrxgene.org/) and Cancer Therapeutics Response Portal (CTRP) (https://portals.broadinstitute.org/ctrp/) databases were used for drug sensitivity analysis (25, 36–40). Finally, the two sub-datasets were pooled, and Pearson’s correlation analyses were performed.

### 2.8 Cell culture

The present study used eight cell lines from four cancers for *in vitro* experiments. Three colorectal cancer cell lines (SW620, LOVO, and HCT116), two lung carcinoma cell lines (A549 and PC9), two liver cancer cell lines (HepG2 and Hep3B), and the cervical cancer cell line Hela are included. HeLa, Hep3B, HepG2, SW620, LOVO, and A549 cells were grown in 10% FBS-supplemented DMEM media. PC9 was grown in 1640 medium containing 10% FBS. HCT116 was grown on McCoy’s 5A medium supplemented with 10% FBS. The cultures were incubated at 37°C with 5% CO2.

### 2.9 Organoids culture

The study was approved by the Ethics Committee of Zhejiang Cancer Hospital, and samples were taken from colorectal cancer patients who underwent surgery at the hospital. After surgery, colorectal samples were sent to the pathology department for pathological examination as part of routine clinical care for cancer patients. Harvesting the tissues had no impact on the patients’ surgical procedures, post-operative radiotherapy or chemotherapy, diagnosis, or the cost of treatment, and therefore the patients’ informed consent was non-mandatory.

A total of 12 colorectal cancer organoids were harvested. Briefly, after obtaining the cancer tissue, the tissue is first thoroughly washed using a washing buffer. The tissue is then cut up and added to the tissue digestion solution. The tumor cells were filtered using a 70 μM filter, resuspended again using the washing buffer, and centrifuged three times. After the removal of the supernatant, the Matrigel (BD, 356234) was added for resuspension. Finally, the cell suspension was inoculated into 48-well plates (Corning 3300). Organoid culture medium purchased from STEMCELL (IntestiCult™ Organoid Growth Medium (Human), Cat.06010).

### 2.10 Drug sensitivity assay

PHA-793887 (HY-11001), PAC-1 (HY-13523), and AZD-7762 (HY-10992) were purchased from MCE (https://www.medchemexpress.com/). DMSO is used as a solvent, and the maximum concentration of DMSO during cell culture does not exceed 0.5%. Organoid viability assay using the CellTiter-Glo^®^ 3D Cell Viability Assay (Promega, G9681). All drug sensitivity verifications were carried out on the third day after the drug was delivered.

### 2.11 Cell viability assay

CCK-8 Cell Counting Kit (A311-01) was purchased from Vazyme (www.vazyme.com/) to assess the proliferative assay. The assay protocol is carried out in accordance with the manufacturer’s manual. The absorbance was measured at 450 nM by a microplate reader (Tecan, Switzerland).

### 2.12 Total RNA extraction and qRT‐PCR

FastPure Cell/Tissue Total RNA Isolation Kit V2 (RC112) from Vazyme^®^ used to extract RNA from cell. HiScript^®^ II Q RT SuperMix for qPCR (+gDNA wiper) (R223) from Vazyme^®^ used to reverse transcription. ChamQ Universal SYBR qPCR Master Mix (Q711) from Vazyme^®^ used for qPCR validation.

Primer of ESPL1 sequences (5’→3’): F: GAAGACTCAGCCTCAGGTG, R: TAGAAAGACCAGTGGCTACG.

Primer of GAPDH sequences (5’→3’): CAGGAGGCATTGCTGATGAT, R: GAAGGCTGGGGCTCATTT.

### 2.13 Cell transfects

siRNA transfect was performed using Lipofectamine 2000 reagent (Invitrogen) according to the manufacturer’s instruction. siRNA-1 sequences: Sense: 5’-AAAGUUGACUCUUUUGAAGCU-3’, Antisense: 5’-CUUCAAAAGAGUCAACUUUGG-3’. siRNA-2 sequences: Sense: 5’-AGACAAAGAGAAUUCGUUCCA-3’, Antisense: 5’-GAACGAAUUCUCUUUGUCUUA-3’.

## 3 Results

### 3.1 Aberrant expression of ESPL1 in cancer tissues

**Figure 1:**
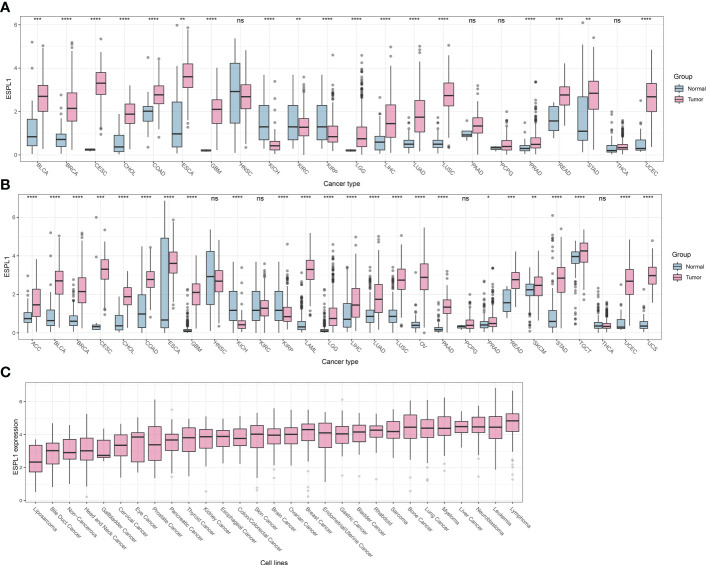
ESPL1 is aberrantly expressed in tumor tissue. **(A)** Expression profile of ESPL1 in TCGA cohorts. **(B)** Expression analysis of ESPL1 in tumor tissues from TCGA database and matched normal tissues from the GTEx database. **(C)** Expression of ESPL1 in different types of cell lines. *P<0.05, **P<0.01; ***P<0.001, ****P<0.0001; ns, Not Significant. GTEx, Data of Genotype-Tissue Expression; TCGA, The Cancer Genome Atlas.

We first compared the difference in expression of ESPL1 between cancer and normal tissues and found that ESPL1 was commonly highly expressed in cancers (**Figure 1A**). Given the insufficient number of normal samples, the data of normal samples from the GTEx database was added for comparison. Results showed that ESPL1 was significantly highly expressed in ACC, BLCA, BRCA, CECS, CHOL, COAD, ESCA, GBM, KIRP, LAML, KICH, LGG, LIHC, LUAD, LUSC, OV, PAAD, PRAD, READ, SKCM, TGCT, STAD, USC and UCEC (**Figure 1B**). We also used the CCLE database to verify cell line-level expression. We found that the highest expression of ESPL1 was in lymphoma, leukemia, neuroblastoma, and liver cancer cell lines (**Figure 1C**). Moreover, the expression of ESPL1 was low in liposarcoma, bile duct cancer, and head and neck cancer cell lines.

Next, we evaluated the level of ESPL1 expression in multiple cancer types at different pathological stages. As shown in **Supplementary Figure 1**, we divided all samples into early (Stage I and II) and late (Stage III and IV) groups based on pathological staging. This type of grouping is more commonly used in clinical trials. We found significantly higher expression of ESPL1 in stage III and IV samples in ACC, CESC, KIPAN, KIRC, KIRP, LIHC, LUAD, UCEC and UCS. In contrast, a different result emerged in THYM and OV, where expression was lower in advanced-stage samples.

Finally, we further validated the dysregulated expression of ESPL1 by comparing it with eight independent GEO datasets, including cervical cancer, lung cancer, liver cancer, and colorectal cancer. Consistently, ESPL1 expression was significantly elevated in cancer samples across all eight datasets (**Supplementary Figure 2**).

We hypothesized that the expression level of ESPL1 may vary in different patients with the same cancer type. Thus, we combined ESPL1 expression with clinical information and found that ESPL1 expression levels showed statistical differences with age and sex in some cancers. As shown in **Supplementary Figure 3A**, the high expression group of ESPL1 had a higher average age in BLCA, KICH, LGG, PRAD, and UCEC, while in BRCA, ESCA, LUSC, LAML, PCPG, and THYM, the high expression group had a lower average age. Similarly, there were gender differences in ESPL1 expression levels, with higher expression levels observed in females in KIRP, LIHC, and SARC, and males had higher expression levels in LAML and LUAD (**Supplementary Figure 3B**).3.2 ESPL1 has potential as a tumor prognostic marker.

Considering that numerous genes highly expressed in cancer tissues affect patient prognosis, we speculated that ESPL1 also impacts patient survival. Therefore, we separated the patients into high and low expression groups for survival analysis based on ESPL1 expression, with the cut-off value by the median of expression.

**Figure 2:**
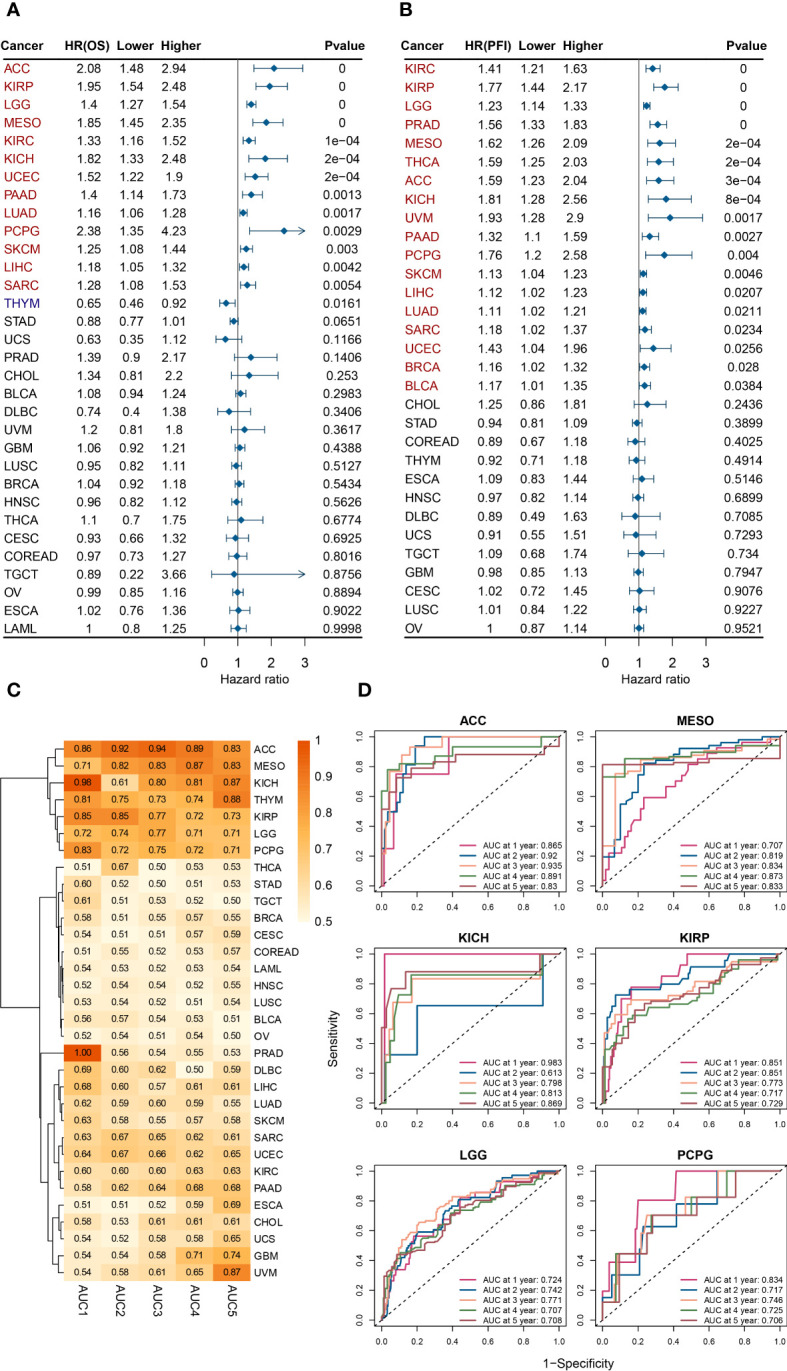
ESPL1 expression correlates with patient prognosis. Forest plot of associations between ESPL1 expression and **(A)** OS and **(B)** PFI. **(C)** Heat map of AUC of ESPL1 expression to predict patient prognosis from 1 to 5 years. **(D)** ROC of ESPL1 expression to predict prognosis in ACC, MESO, KICH, KIRP, LGG and PCPG. OS, overall survival; PFI, Progression Free Interval; AUC, Area Under Curve; ROC, Receiver Operating Characteristic.

As shown in **Figure 2**, a univariate analysis was performed with patient death as the event endpoint. Results showed that the prognosis of patients was worse in the ESPL1 high expression group in ACC, KIRP, LGG, MESO, KIRC, KICH, UCEC, PAAD, LUAD, PCPG, SKCM, LIHC, and SARC. Conversely, a positive correlation was found between high ESPL1 expression and improved prognosis in THYM. In addition, it was found that ESPL1 was highly expressed in ACC, CHOL, KIRC, KIRP, LGG, LUAD, SKCM, and UCEC cancerous tissues, and it shortened the survival of patients. **Figure 2B** demonstrates the relationship between ESPL1 expression and Progression Free Interval (PFI), where we found that in 18 types of cancer, high expression of ESPL1 was associated with poorer PFI.

Multivariate analysis is a statistical technique that analyzes the relationships between multiple variables in a dataset. It is used to determine the strength and direction of the relationships between variables, and to identify patterns and trends in the data. We combined ESPL1 expression with various clinical information and verified the effect of ESPL1 on patient prognosis through multivariate analysis. As shown in **Supplementary Figure 4**, ESPL1 remained a prognostic risk factor (HR>1 and p<0.05) for ACC, KICH, LUAD, MESO, PAAD, PCPG, SKCM, SARC, and LGG, further suggesting that ESPL1 may play an oncogenic role.

We postulated that ESPL1 could potentially serve as a marker for predicting cancer development. We constructed a receiver operating characteristic curve based on ESPL1 expression to test this. As shown in **Figure 2C**, the heat map demonstrates the area under the curve (AUC) for predicting patient OS for ESPL1 in 32 tumors. In ACC, MESO, KICH, KIRP, LGG, and PCPG, the AUC were determined with high precision to be greater than 0.70 (**Figure 2D**). Specifically, in ACC, the AUC of ESPL1 predicted prognosis with a value between 0.83 and 0.94. In GBM and UVM, the AUC for predicting 5-year survival reached 0.74 and 0.87, respectively, although the accuracy of predicting 1-4 year prognosis was poor.

**Figure 3:**
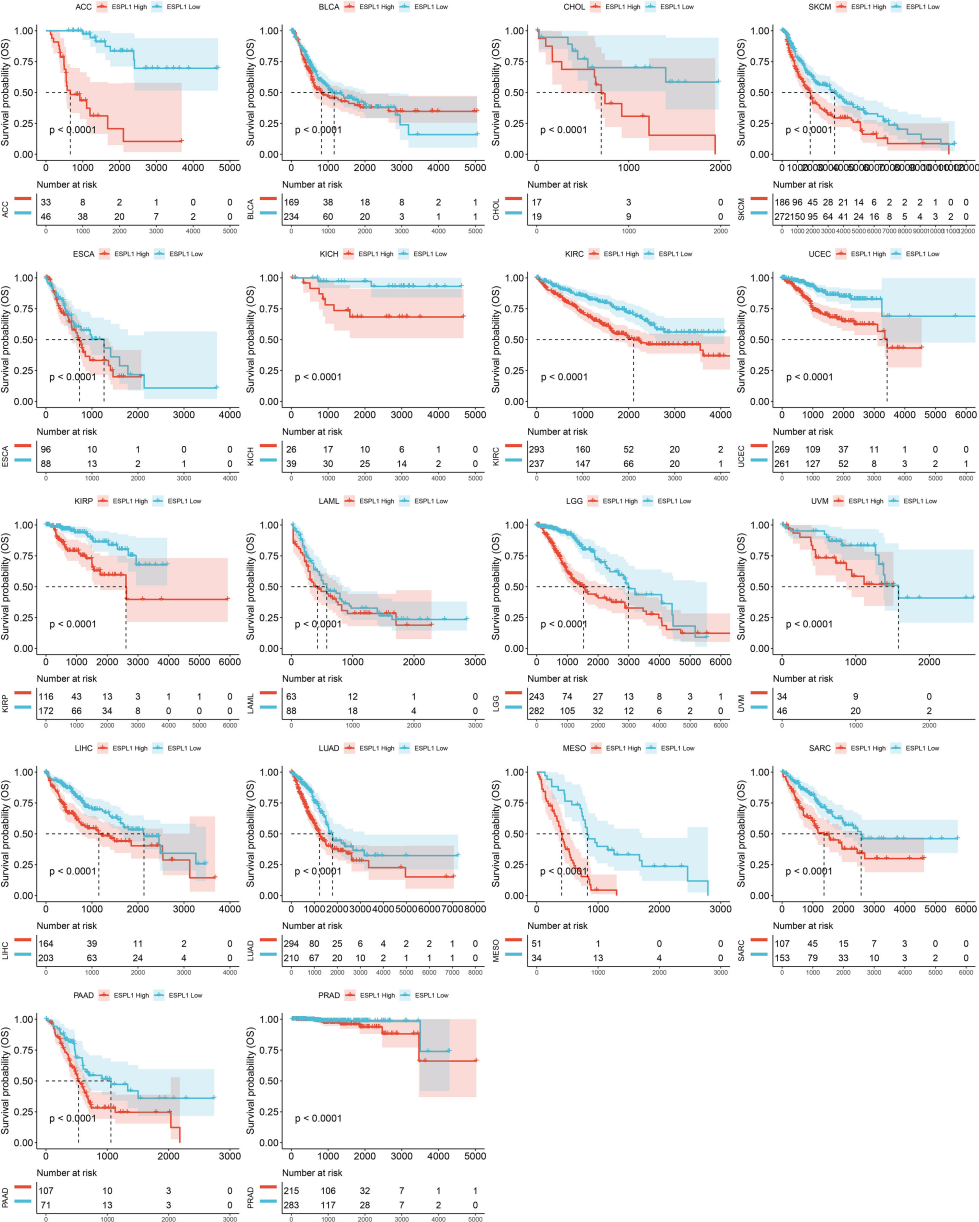
Kaplan-Meier plots with statistically significant differences in overall survival analysis by best-cut off value method for ESPL1.

Finally, we plotted Kaplan-Meier survival curves grouped according to ESPL1 expression based on the best cut-off value method. As shown in **Figure 3**, the survival time was shorter for high expression of ESPL1 in the 18 tumors.

Based on these findings, we conclude that ESPL1 may have oncogenic characteristics, and high expression is associated with poorer prognosis in cancer patients. We believe that, following validation through further prospective clinical studies, ESPL1 has the potential to become a prognostic biomarker in various malignancies.

### 3.3 Correlation between ESPL1 and tumor microenvironment

**Figure 4:**
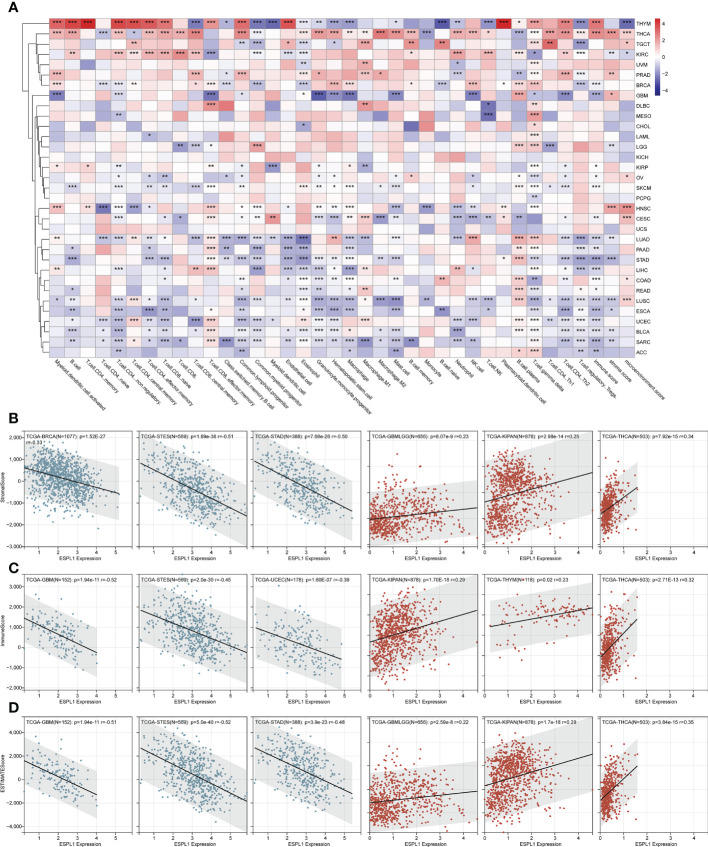
The effect of ESPL1 on TME in pan-cancers. **(A)** Correlation between ESPL1 and TME cells by xCELL algorithm. Representative results of correlation analysis between ESPL1 expression and immune score **(B)**, stromal score **(C)** and ESTIMATE score **(D)** by ESTIMATE algorithm (Three most positive correlations versus three most negative correlations). *p<0.05, **p<0.01, ***p<0.001. TME, tumor microenvironment.

The tumor microenvironment (TME) is critical for tumor growth and is directly associated with tumor progression and metastasis. Therefore, we analyzed the correlation between ESPL1 expression in various cancers and the immune cells/scores using the XCELL algorithm (**Figure 4A**). We found that THYM and THCA correlated extremely well with ESPL1 expression. In particular, in THYM, there was a strong correlation with a variety of T cells. In contrast, in THCA, ESPL1 expression was positively correlated with immune cells and stromal cells. LUAD, PAAD, STAD, LIHC, COAD, LUSC, ESCA, UCEC, BLCA, and SARC negatively correlated with immune microenvironment cells. This result gives us a hint that ESPL1 may play different roles in the immune microenvironment in different cancers.

We also calculated the immune, stromal, and ESTIMATE scores using the ESTIMATE algorithm. It was found that the expression of ESPL1 was negatively correlated with these scores in most cancers. However, THCA, KIPAN, GBMLGG, and KIRC were positively correlated with immune scores (**Figure 4B**; **Supplementary Table 2**). Similarly, THCA, KIPAN, and GBMLGG were positively correlated with stromal scores (**Figure 4C**; **Supplementary Table 2**). The same results were also found about the estimate scores. Moreover, the expression of ESPL1 was positively correlated with the ESTIMATE score in THCA, KIPAN, GBMLGG, and KIRC (**Figure 4D**; **Supplementary Table 2**). In THYM, ESPL1 was significantly positively correlated with the immune score but negatively correlated with the ESTIMATE score. This finding is consistent with xCell results, which show that ESPL1 expression significantly correlates with T and B cells, increasing the immune score. However, the correlation with stromal cells is negative or not significant, resulting in a negative correlation in stromal score. In LUAD, PAAD, STAD, LIHC, COAD, READ, LUSC, ESCA, UCEC, BLCA, SARC, and ACC, the xCell results demonstrated a negative correlation trend between various T cells, B cells, macrophages, and ESPL1 expression, which is consistent with the immune score in ESTIMATE. Overall, the three immune scores showed a significant negative trend in GBM, ESCA, STES, SARC, STAD, UCEC, SKCM, PAAD, OV, BLCA, and ACC. In KIPAN, THCA, there was a significant positive correlation. No statistically significant correlations existed in MESO, READ, KIRP, LAML, UVM, UCS, CHOL, and DLBC. This indicates that the function of ESPL1 may differ significantly among different types of tumors.

### 3.4 Correlation between ESPL1 expression and immune markers

**Figure 5:**
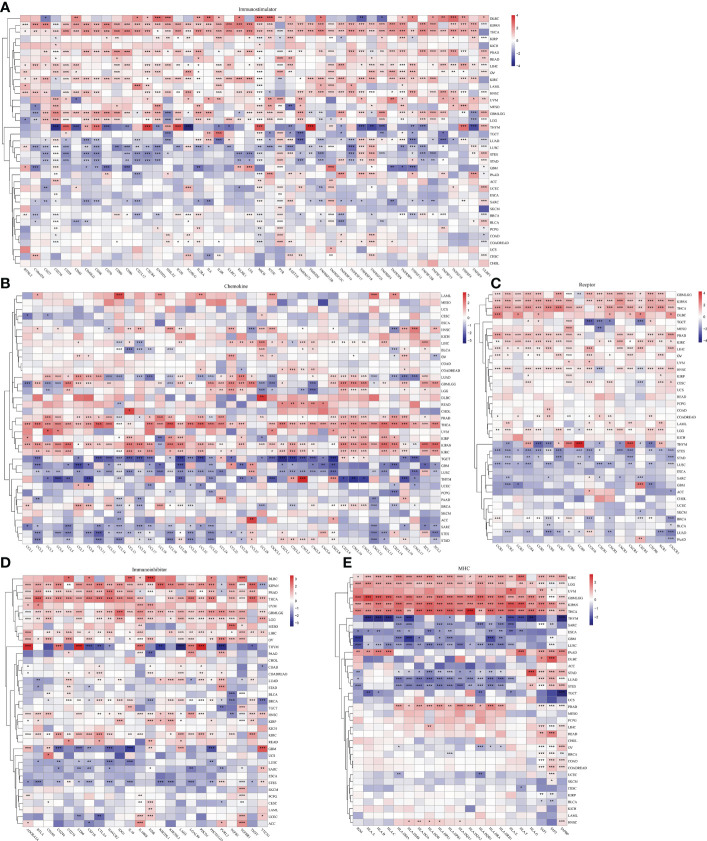
The effect of ESPL1 on immunological genes in pan-cancers. Correlation between ESPL1 and **(A)** immunostimulators, **(B)** chemokines, **(C)** receptors, **(D)** Immunoinhibitor and **(E)** MHC. *p<0.05, **p<0.01, ***p<0.001.

Given that immunoregulatory genes are closely associated with cancer development, we evaluated the expression data of 150 immunoregulatory genes in each sample and correlated them with the expression of ESPL1 (**Figures 5A–E**). **Figure 5A** shows the heatmap of immunostimulatory genes with ESPL1 expression. Through clustering, we found high positive correlations in DLBC, KIPAN, and THCA. While in PRAD, READ, LIHC, OV, KIRC, LAML, HNSC, UVM, MESO, and GBMLGG, there is a predominantly positive correlation trend. An extremely strong correlation emerged in THYM. This trend switched to a negative correlation in LUAD, LUSC, and STES. Notably, CD276, MICB, PVR, and ULBP1 showed statistically significant correlations with ESPL1 in most tumors, suggesting that these genes may be essential to unlocking the influence of ESPL1 on tumor development. Chemokines are very powerful and can impact tumor migration and immune cell infiltration. Through **Figure 5B**, we explored the correlation between chemokines and ESPL1. Similarly, the correlations showed a divergent trend, with the expression of chemokine genes increasing with the expression of ESPL1 in KIPAN, KIRC, THCA and, conversely, a statistically negative correlation in TGCT, GBM, LUSC and THYM. **Figures 5C–E** shows the correlation results of ESPL1 with receptor, immunoinhibitor and MHC, respectively. The bifurcation trend was again observed, with STES, STAD, and LUSC showing a negative trend among receptor-related genes, while GBMLGG, KIPAN, THCA, PRAD, KIRC, LIHC, and HNSC showed a positive trend. In the correlation analysis with MHC, significant positive correlations were also found in KIRC, LGG, GBMLGG, KIPAN, THCA, and PRAD. These results suggest that the correlation between ESPL1 and immunity is extremely strong in KIPAN, GBMLGG, and THCA; in these tumors, more immune-related validation is needed.

### 3.5 Correlation between ESPL1 expression and tumor heterogeneity

**Figure 6:**
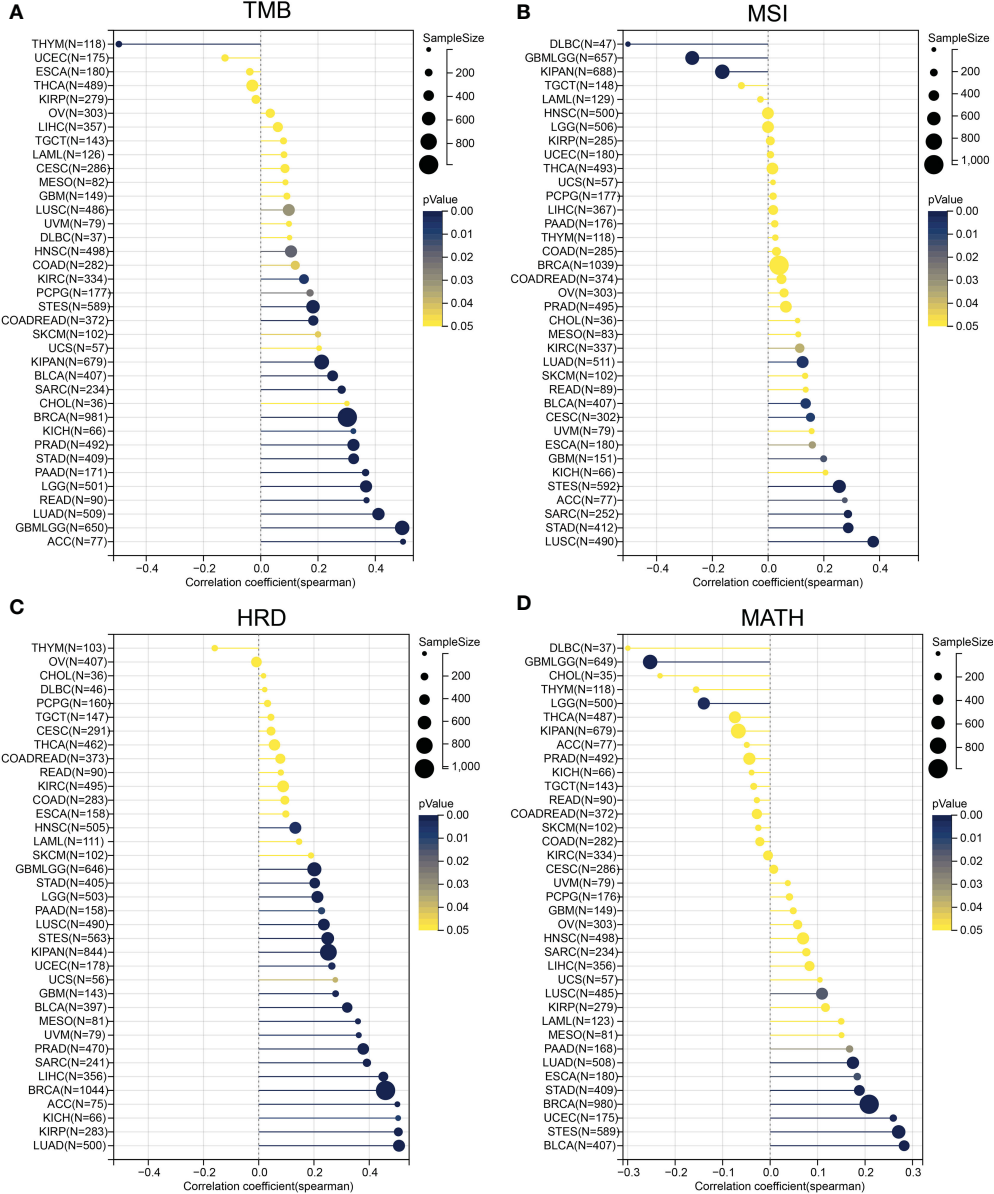
Correlation of ESPL1 with tumor heterogeneity. Correlation between ESPL1 expression and **(A)** TMB, **(B)** MSI, **(C)** HRD and **(D)** MATH. TMB, tumor mutational burden; MSI, microsatellite instability; HRD, homologous recombination deficiency; MATH, mutant-allele tumor heterogeneity.

Considering that TMB and MSI correlated with immunotherapy efficacy, we further assessed the correlation between ESPL1 expression and TMB and MSI. Immune checkpoint inhibitor sensitivity is associated with high tumor mutational burden (TMB), **Figure 6A** shows the information of TMB with ESPL1 expression in each cancer. The results indicated that TMB positively correlated with ESPL1 expression in DLBC, CHOL, ACC, LUAD, KICH, PRAD, LGG, STAD, PADD, BRCA, SARC, and READ. Surprisingly, there was a statistically negative correlation between the expression of ESPL1 and TMB in THYM, with high expression of ESPL1 being associated with a better prognosis. The instability of microsatellites results from defects in the mismatch repair system, resulting in hypermutation patterns. MSI is often used to guide treatment, such as in colorectal cancer, where immune checkpoint blockade treatment decisions are made based on a patient’s MSI status. From **Figure 6B**, we can find that MSI showed a significant negative correlation with the expression of ESPL1 in DLBC and a positive trend in LUSC, ACC, and STAD. Previous studies have reported that homologous recombination deficiency will produce specific and quantifiable genomic changes, and the HRD status is a key indicator of treatment and prognosis in many tumors (21, 41, 42). After analyzing the relationship between HRD and ESPL1 expression, we found that HRD increased with the increase of ESPL1 expression in 22 types of tumors (**Figure 6C**). Mutant-allele tumor heterogeneity (MATH) is an algorithm for assessing tumor heterogeneity, with higher MATH values indicating higher tumor heterogeneity (26, 43). This study explored the relationship between MATH and ESPL1 expression and found a significant correlation in 14 tumors, with a positive correlation in 10 tumors and a negative correlation in four tumors (GBMLGG, LGG, KIPAN, and THCA) (**Figure 6D**).

### 3.6 Enrichment analysis of ESPL1

**Figure 7:**
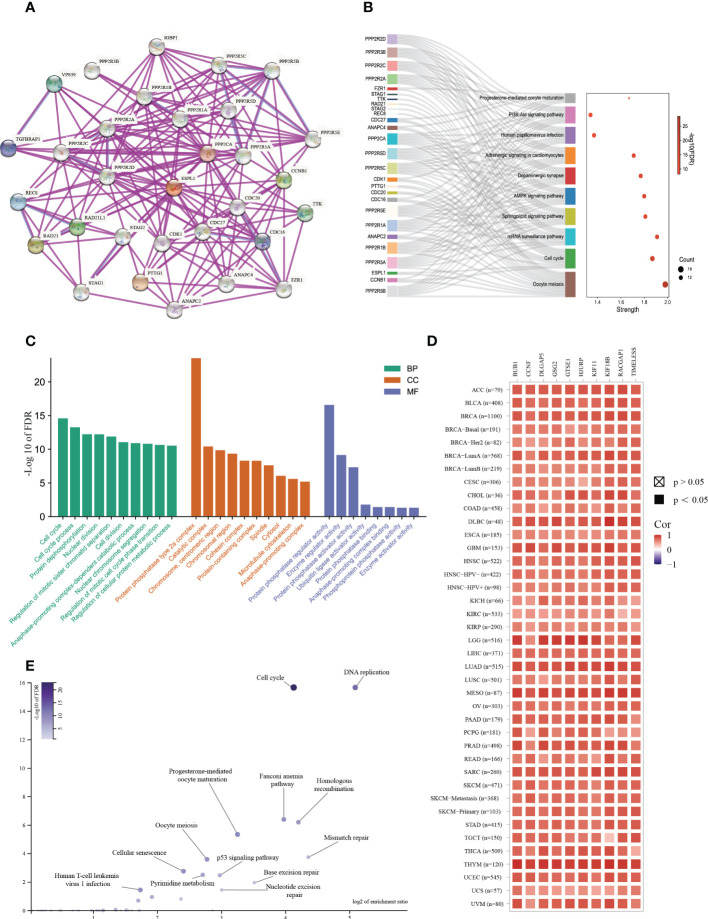
Enrichment analysis of ESPL1-related partners. **(A)** PPI analysis of ESPL1. **(B)** Sankey diagram of KEGG pathway analysis results. **(C)** GO functional classification. **(D)** Heat map of ESPL1 correlations with the top 10 similar genes in different cancer types. **(E)** Volcano plot of KEGG results for 200 similar genes. PPI, protein-protein interaction; KEGG, Kyoto Encyclopedia of Genes and Genomes. BP, biological process. CC, cellular component. MF, molecular function. FDR, false discovery rate.

To further explore the molecular mechanisms and functions of the ESPL1 gene in tumorigenesis, enrichment analysis was performed to screen for ESPL1-related proteins and pathways. First, protein–protein interaction (PPI) network analysis was performed using STRING, and the top 30 genes associated with ESPL1 were obtained (**Figure 7A**). After KEGG analysis of these genes and drawing Sangchi map, it was found that the pathways significantly associated with tumor were enriched in cell cycle, the AMPK signaling pathway, and the PI3K Akt signaling pathway (**Figure 7B**). Next, we performed gene ontology (GO) enrichment analysis with regard to biological processes, cell components, and molecular functions (**Figure 7C**). In addition, we combined the expression data of all TCGA tumors and identified the top 200 genes most related to ESPL1 expression (the list of the top 200 similar genes is provided in **Supplementary Table 3**). We then analyzed the correlation between the first ten similar genes and ESPL1 and found that all were significantly positively correlated with ESPL1 expression (**Figure 7D**). Moreover, the KEGG enrichment results showed that the 200 genes were mainly associated with cancer-related pathways, including cycle, DNA replication, and mismatch repair (**Figure 7E**). To further explore the biological functions of down-regulated ESPL1-related proteins, we obtained the top 200 genes negatively correlated with the expression level of ESPL1. We performed enrichment analysis on these genes (**Supplementary Tables 4**, **5**). However, these 200 genes showed no statistically significant enrichment in the terms or pathways identified in the KEGG or GO analyses. No terms with an FDR<0.05 were enriched for BP, MF, and CC. This set of 200 genes may have lacked annotations in the enrichment analysis or may not be involved in any specific biological functions.

### 3.7 Drug sensitive analyses of ESPL1

**Figure 8:**
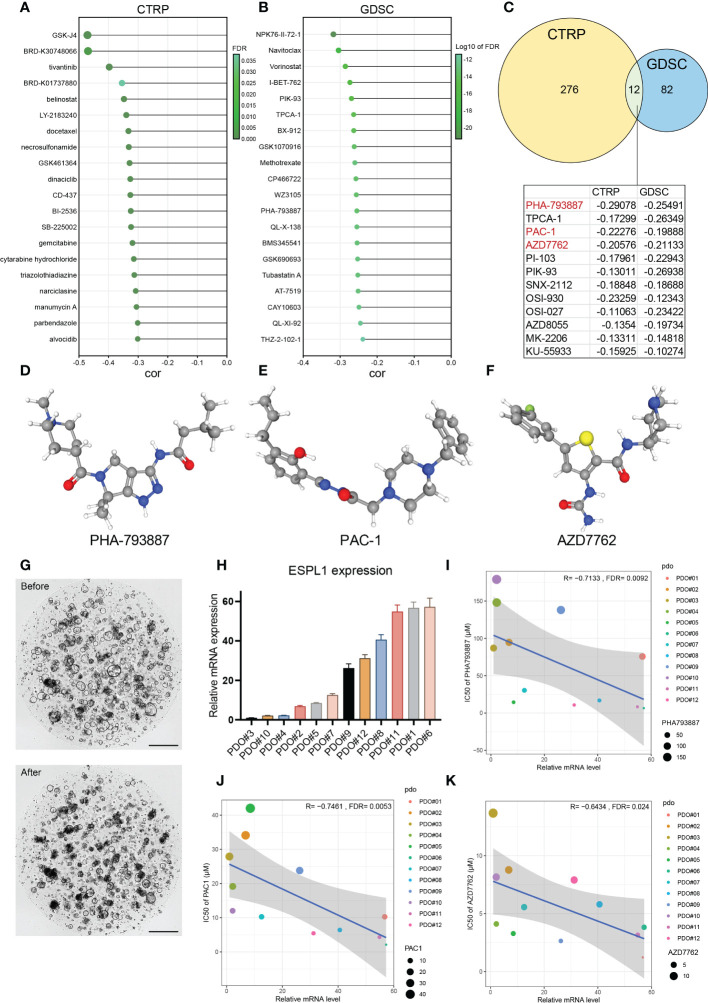
The relationship between ESPL1 and drug sensitivity. The drugs with the strongest correlation in ESPL1 expression were in the **(A)** CTRP and **(B)** GDSC databases. **(C)** Venn diagram of the results of the two databases. **(D-F)** The three-dimensional structure of drugs in PubChem. **(G)** Patient derived organoids (PDO) before and after coculture with drugs. Scale with 500 μM. **(H)** Expression level of ESPL1 in 12 PDOs. Correlation of ESPL1 expression in organoids with IC50 of **(I)** PHA793887, **(J)** PAC-1 and **(K)** AZD7762.

Furthermore, GDSC and CTRP, two of the largest tumor-related drug databases, were utilized to discover drugs that target tumors with high ESPL1 expression. the CTRP database indicated that GSK-J4 (R= -0.471) and BRD-K30748066 (R= -0.469) were the most negatively correlated with ESPL1 expression (**Figure 8A**). **Figure 8B** shows the top 20 drugs negatively correlated with high ESPL1 expression *via* GDSC, with NPK76-II-72-1 (R= -0.318) being the most negatively correlated. Using the intersection, we identified twelve medicines that appeared in both datasets (**Figure 8C**). Three medications that impede the cell cycle or induce apoptosis are among the most remarkable findings from comparing the data. PHA-793887 is a strong CDK inhibitor with anti-cancer effects on the cell cycle (44). The activation of procaspase-3, which promotes apoptosis, is the approach through which Procaspase activating compound 1 (PAC1) kills cancer cells (45). AZD-7762 is a checkpoint kinase inhibitor that inhibits tumor proliferation and growth by targeting Chk1 and Chk2 (46). Coincidentally, ESPL1 is a critical cell cycle regulator, and as ESPL1 expression rises, so does the drug sensitivity of the cell cycle inhibitors list above.

Therefore, we determined the connection between ESPL1 expression and these three small molecule inhibitors using colorectal cancer patient-derived organoids. **Figures 8D–F** show the molecular structures from PubChem of PHA-793887, PAC1, and AZD-7762, respectively. **Figure 8G** depicts images of the organoids in normal culture before and two days after adding the drugs (days 0 and 2). The normal growth of the organoid had a circular form with a maximum diameter of 200 μM; however, the addition of drugs resulted in a considerable reduction in roundness, fragmentation, and darkening. **Figure 8H** shows the distribution of the expression of ESPL1 in 12 cases of organoid. The expression of ESPL1 varied greatly, with the maximum expression of PDO#6 being 57 times higher than the lowest expression. By analyzing the link between drug sensitivity and ESPL1 expression in the organoids, we determined that the IC50 of the three drugs reduced dramatically with increasing ESPL1 expression (**Figures 8I–K**). We also discovered that the IC50 of PHA-793887 varied widely between organoids, with the greatest IC50 reaching 179 μM (PDO#10) and the lowest reaching only 6.6 μM (PDO#06).

To investigate whether ESPL1 is a direct target of PAC1, AZD-7762, and PHA-793887, we performed IC50 assays after knocking down ESPL1 expression in HCT116 and SW620 cell lines. As shown in the **Supplementary Figure 5**, there was no significant change in the IC50 values of the three drugs after ESPL1 knockdown. Only in SW620, the IC50 of AZD7762 was reduced after knockdown using si1, which was unexpected and may contradict our initial hypothesis.

### 3.8 Knockdown of ESPL1 impact on proliferation *in vitro*

**Figure 9:**
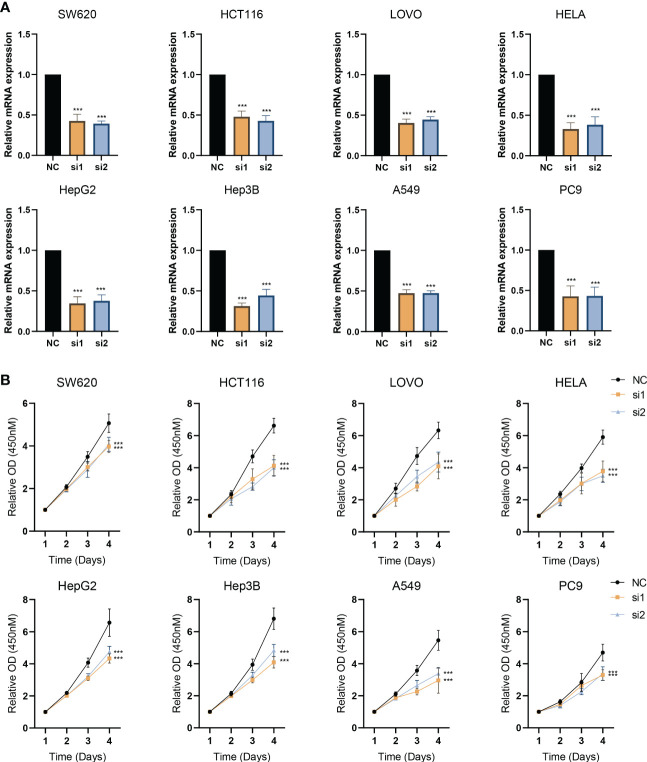
Interference with ESPL1 expression inhibits cell proliferation in a variety of cell lines. **(A)** Validation of siRNA interference efficiency. **(B)** Cell proliferation curves following interference with ESPL1 expression in eight different cell lines.

A total of 8 types of cell lines, including colorectal, liver, lung, and cervical cancer, were used to verify the impact of knockdown ESPL1. ESPL1 is highly expressed in these cancers, and the prognosis is worse for high expression. Initially, we inhibited the expression of ESPL1 in these cell lines using siRNA and confirmed the results at the mRNA level (**Figure 9A**). We found that cell proliferation in both cancer cell lines was significantly inhibited following interference with ESPL1 expression (**Figure 9B**). This conclusion is consistent with expectations, as ESPL1 is a critical gene involved in cell division, and its suppression has a definite effect on cell proliferation.

## 4 Discussion

The cell cycle represents a series of tightly integrated events that allow the cell to grow and proliferate (27). Notably, cancer represents a dysregulation of the cell cycle so cells that overexpress cyclins or do not express the CDK inhibitors continue to undergo unregulated cell growth (27, 47). ESPL1 encodes separase, a protein that regulates the cell cycle and plays an important role in the process of chromosome segregation. Previous studies have confirmed that ESPL1 is an oncogene that is overexpressed in many human cancers of breast, bone, brain, and prostate (48, 49). However, although researchers have gained some insight into the cell cycle regulation by ESPL1, more is needed to know whether and how it drives tumorigenesis, progression, and metastasis.There are no relevant pan-cancer analyses to date. Overall, as a key cell cycle-associated gene, the potential role of ESPL1 in carcinogenesis and cancer development is worth investigating.

First, we investigate the relationship between ESPL1 expression and the prognosis for survival of common cancers. Comparing cancer tissues to normal tissues revealed that ESPL1 was highly elevated in a number of malignancies. This could be due to the fact that upregulation of ESPL1 promotes cell cycle progression, resulting in a rapid increase in cell proliferation. Moreover, by comparing the expression of ESPL1 in various clinical stages, we discovered that ESPL1 expression increased as pathological stages progressed. Interestingly, as the disease advanced in SKCM and OV, ESPL1 expression decreased, particularly in SKCM, where patients with high ESPL1 expression had a poorer prognosis. However, the tumor stage was negatively correlated with the expression of ESPL1, a phenomenon that deserves further study. Kaplan-Meier and univariate Cox regression analyses revealed that upregulation of ESPL1 expression was associated with poor prognosis. Using the optimal cutoff value, we found that high expression of ESPL1 was significantly associated with poor prognosis in 18 different types of cancer. To avoid sample size imbalance, we ensured that the sample size of each group was at most 60% of the total sample size after grouping, thus ensuring comparability and statistical significance between the two groups. However, high ESPL1 expression was associated with better OS prognosis in THYM patients, implying that ESPL1 may be protective in this cancer. However, the PFI of THYM predicted by ESPL1 did not statistically distinguish a better prognosis, suggesting that additional confounding factors influenced the prediction of OS by ESPL1 in THYM. Through multivariate analysis that integrates clinical information, ESPL1 remains a prognostic risk factor in multiple types of cancer. We hypothesized that ESPL1 expression is a reliable indicator of prognosis. Using ROC, we obtained an AUC of 0.7+ in ACC, MESO, KICH, KIRP, LGG, PCPG, GBM, THYM, and UVM for predicting 5-year survival.

It is worth noting that cancers develop in complex tissue environments, the tumor microenvironment, which they depend upon for sustained growth, invasion, and metastasis (50). TME consists of three critical components: tumor cells, stromal cells, and ECM (51). This study also integrated, for the first time, the correlation between ESPL1 expression and the tumor microenvironment. Results demonstrated that high expression of ESPL1 in THYM showed a positive correlation with various CD4+ T cells, and a negative correlation with epithelial cells and macrophages. However, most other cancers showed a negative correlation with CD4+ T cells, which may be one of the reasons why the high expression of ESPL1 in THYM exhibited a better prognosis. Moreover, we found that Th2 cell was positively correlated with ESPL1 expression in the majority of tumors.

The ESTIMATE algorithm has been shown to predict tumor purity and reflects the characteristics of TME. Most tumor scores decreased with the increase in expression of ESPL1, but the opposite was true for THCA. The three scores were positively correlated with ESPL1 expression, and most cells in the TME were positively correlated with ESPL1 expression; in THCA, ESPL1 may affect immunity through a different mechanism. The immune score reflects the number and functional status of immune cells infiltrating the tumor microenvironment, including T cells, B cells, plasma cells, natural killer cells, and others. By using Immune Score, we can obtain information about the immune infiltration in the tumor microenvironment. XCELL, on the other hand, provides a detailed evaluation of each type of immune cell present in the microenvironment. In summary, these two algorithms can help us understand the relationship between ESPL1 and the tumor microenvironment from a macro and cellular level.

A slight association between TME cells and ESPL1 expression was found in UCS and CHOL, which implies that ESPL1 is not a suitable TME therapeutic target in these two tumors.

The same conclusion was obtained in the pan-correlation analysis, which explored the association between immune-related genes and ESPL1. The analysis showed that the correlation between ESPL1 and immune-related genes in USC and CHOL was not strong, suggesting that the effect of ESPL1 on these two cancers is not through the immune function. CD276 belongs to the immunoglobulin superfamily and participates in the regulation of T-cell-mediated immune response. We found that CD276 is statistically correlated with ESPL1 in a variety of tumors and that there may be an intrinsic link between them. ULBP1 is a ligand of NKG2D, an immune system-activating receptor on NK cells and T-cells. here was also a significant co-expression relationship between ESPL1 and ULBP1; ESPL1 could be involved in the immune regulation of tumors. Notably, ESPL1 was negatively correlated with immune-related genes in LUAD, LUSC, STAD, THYM, SARC, GBM, and TGCT. High expression of ESPL1 was associated with better survival in THYM, suggesting that ESPL1 may influence patient prognosis by affecting immunity.

The TMB, MSI, MATH, and HRD are indicators of tumor heterogeneity and can be used to guide application of tumor immunotherapy. In THYM, the expression of ESPL1 was negatively correlated with TMB, while high expression of ESPL1 was coincidentally associated with a better prognosis. This may indicate that ESPL1 could decrease TMB and thus improve patient survival, but further validation is required. In BLCA, STAD, and LUSC, ESPL1 expression was positively correlated with TMB and MSI, suggesting that these tumors may show good response to immunotherapy. In LUSC and BLCA, ESPL1 was also positively correlated with MATH, HRD, and all the four indicators suggesting that target ESPL1-targeted treatments may be effective in LUSC and BLCA.

Analysis of GDSC and CTRP databases identified drugs negatively correlated with ESPL1, suggesting that tumor cells with high ESPL1 expression are likely to be more sensitive to these drugs. GSK-J4 is a potent dual inhibitor of H3K27me3/me2-demethylases JMJD3/KDM6B and UTX/KDM6A. GSK-J4 inhibits LPS-induced TNF-α production in human primary macrophages and can induce endoplasmic reticulum stress-associated apoptosis. GSK-J4 is thought to be effective in diffuse intrinsic pontine glioma (DIPG) (52), and the drug sensitivity of GSK-J4 is enhanced with increased expression of ESPL1. Perhaps it is feasible to use ESPL1 as an indication for GSK-J4 in DIPG. BRD-K30748066 is a CDK9 inhibitor, a member of the cyclin-dependent protein kinase (CDK) family. This correlation is consistent with the function of ESPL1. A total of 12 drugs were shown to be more sensitive in cancer cell lines with high ESPL1 by correlation analysis of the two drug databases. Interestingly, a variety of mTOR inhibitors were involved, including AZD-8055, OSI-027 and PI-103. In addition, pro-apoptotic and cell cycle inhibiting drugs are also listed, including PHA-793887, PAC-1 and AZD-7762. Through organoid drug sensitivity testing, we confirmed that the expression of ESPL1 was statistically linked with PHA-793887, PAC-1, and AZD-7762, and that the expression of ESPL1 in colorectal cancer patient tissues may indicate the use of these drugs. In patients with high ESPL1 expression, certain drugs may be more effective. However, interference with ESPL1 expression in cell lines should lead to increased drug resistance. The absence of this trend may indicate that ESPL1 does not directly affect the response to these three drugs. The relationship and mechanisms between ESPL1 expression levels and PAC1, AZD7762, and PHA793887 deserve further investigation and discussion.

Finally, through *in vitro* studies, we demonstrated that ESPL1 can impact the proliferation, which is concordant with the bioinformatics results.

In conclusion, this study demonstrates the potential of ESPL1 as a cancer biomarker in various malignancies, with high expression of ESPL1 associated with worse prognosis in multiple cancer types and immune infiltration. Additionally, ESPL1 expression is associated with TMB, MSI, MATH, and HRD in several cancer types, suggesting a connection with tumor heterogeneity. We assessed drug sensitivity using organoids and found that those with high ESPL1 expression were more vulnerable to cell cycle inhibitors. Therefore, ESPL1 could serve as a marker for cancer therapy. *In vitro* assays confirmed that interference with ESPL1 can affect cell proliferation. Nonetheless, the study has some limitations, including the small sample size for organoid drug sensitivity tests, which may lead to bias. Future research should further investigate ESPL1 in other malignancies.

## 5 Conclusions

Through the use of public data mining, we were able to confirm that ESPL1 is an oncogene, that it can serve as a prognostic marker for several cancers, that it can be used to direct cancer medication therapy in patient derived organoids, and that ESPL1 knockdown can limit cell growth *in vitro*.

## Data availability statement

The original contributions presented in the study are included in the article/**Supplementary Material**. Further inquiries can be directed to the corresponding authors.

## Ethics statement

The studies involving human participants were reviewed and approved by the Ethics Committee of Zhejiang Cancer Hospital. Written informed consent for participation was not required for this study in accordance with the national legislation and the institutional requirements.

## Author contributions

YZ conceived the idea, analyzed the data, and drafted the work. CZ and YZ analyzed the data and performed the visualization. WZ, HW, MW, and QZ collected the data and participated in the revision. HF and GW supervised the study. HF and GW provide funding support. All authors contributed to the article and approved the submitted version.

## Supplementary material

Supplementary Figure 1 Relationship between ESPL1 expression and pathological staging. ns. not significant; *, p<0.05; **, p<0.01; ***, p<0.001.

Supplementary Figure 2 Validating the aberrant expression of ESPL1 through GEO. **(A, B)** Cervical cancer. **(C, D)** Liver cancer, **(E, F)** Lung cancer, **(G, H)** Colorectal cancer.

Supplementary Figure 3 Relationship between ESPL1 expression and clinical information. **(A)** Box plots showing the relationship between ESPL1 expression and age. **(B)** Box plots showing the relationship between ESPL1 expression and gender.

Supplementary Figure 4 Multivariate survival analysis based on ESPL1 expression and multiple clinical information.

Supplementary Figure 5 The IC50 values of HCT116 and SW620 cells after siRNA-mediated interference of ESPL1 expression. (A and C) IC50 curves of three drugs in HCT116 and SW620 cells. (B and D) Column chart comparing IC50 values.
