# Supplementary figures and images for: Pan-Cancer analysis and experimental validation identify the oncogenic nature of ESPL1: Potential therapeutic target in colorectal cancer
Source: Front Immunol. 2023 Mar 16;14:1138077. doi: 10.3389/fimmu.2023.1138077 (PMC10060535; doi:10.3389/fimmu.2023.1138077)

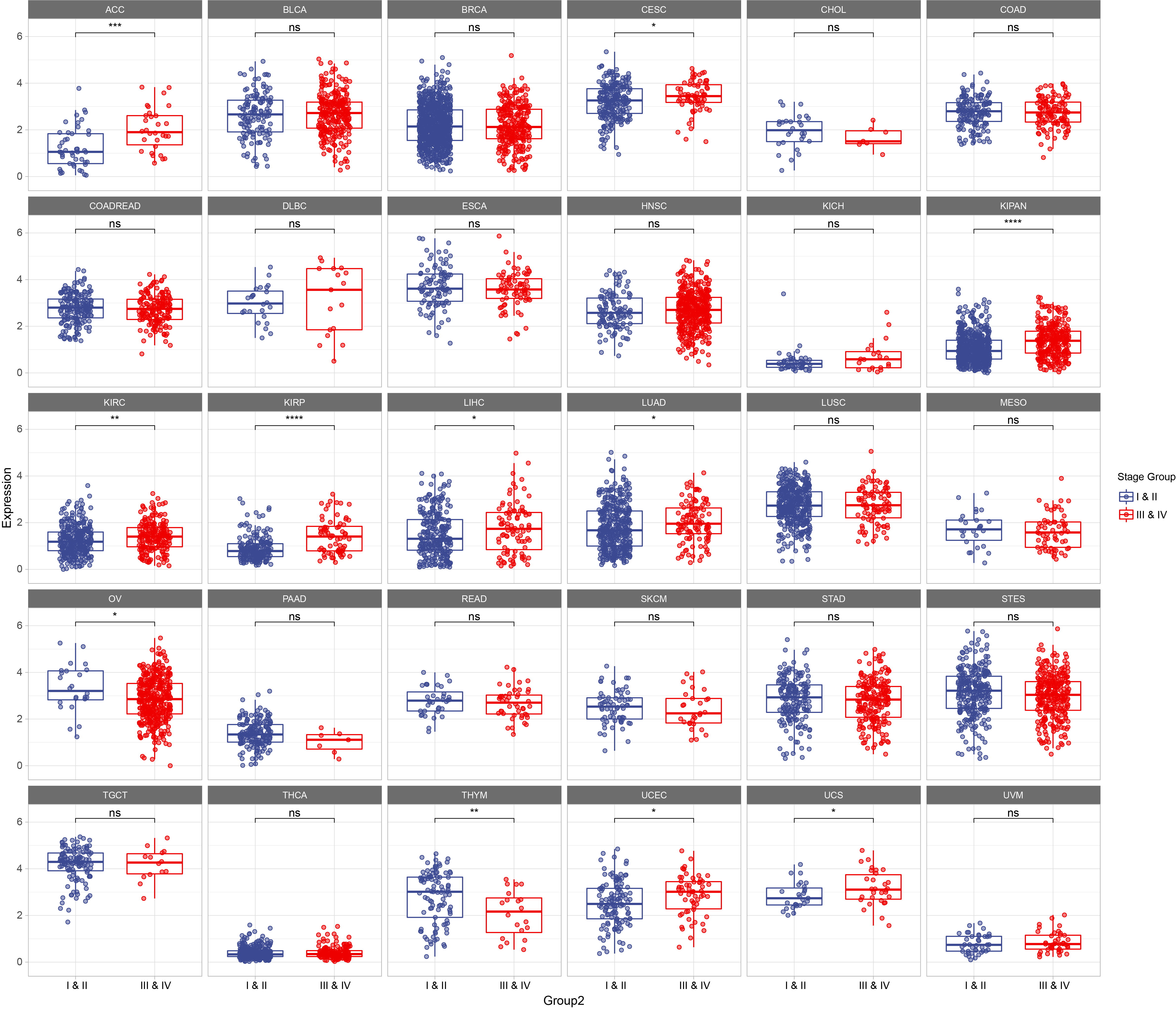

Supplement: Supplementary Figure 1 — Relationship between ESPL1 expression and pathological staging. ns. not significant; *, p<0.05; **, p<0.01; ***, p<0.001. [file Image_1.jpeg]

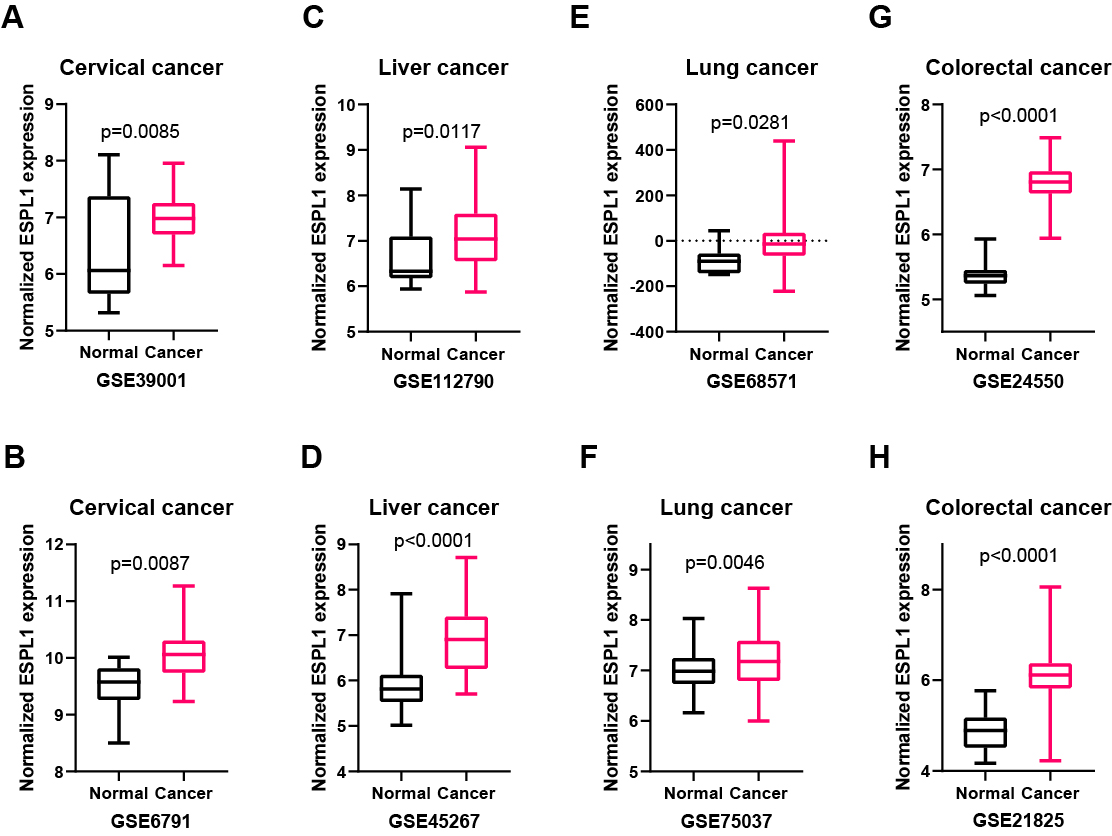

Supplement: Supplementary Figure 2 — Validating the aberrant expression of ESPL1 through GEO. (A, B) Cervical cancer. (C, D) Liver cancer, (E, F) Lung cancer, (G, H) Colorectal cancer. [file Image_2.jpeg]

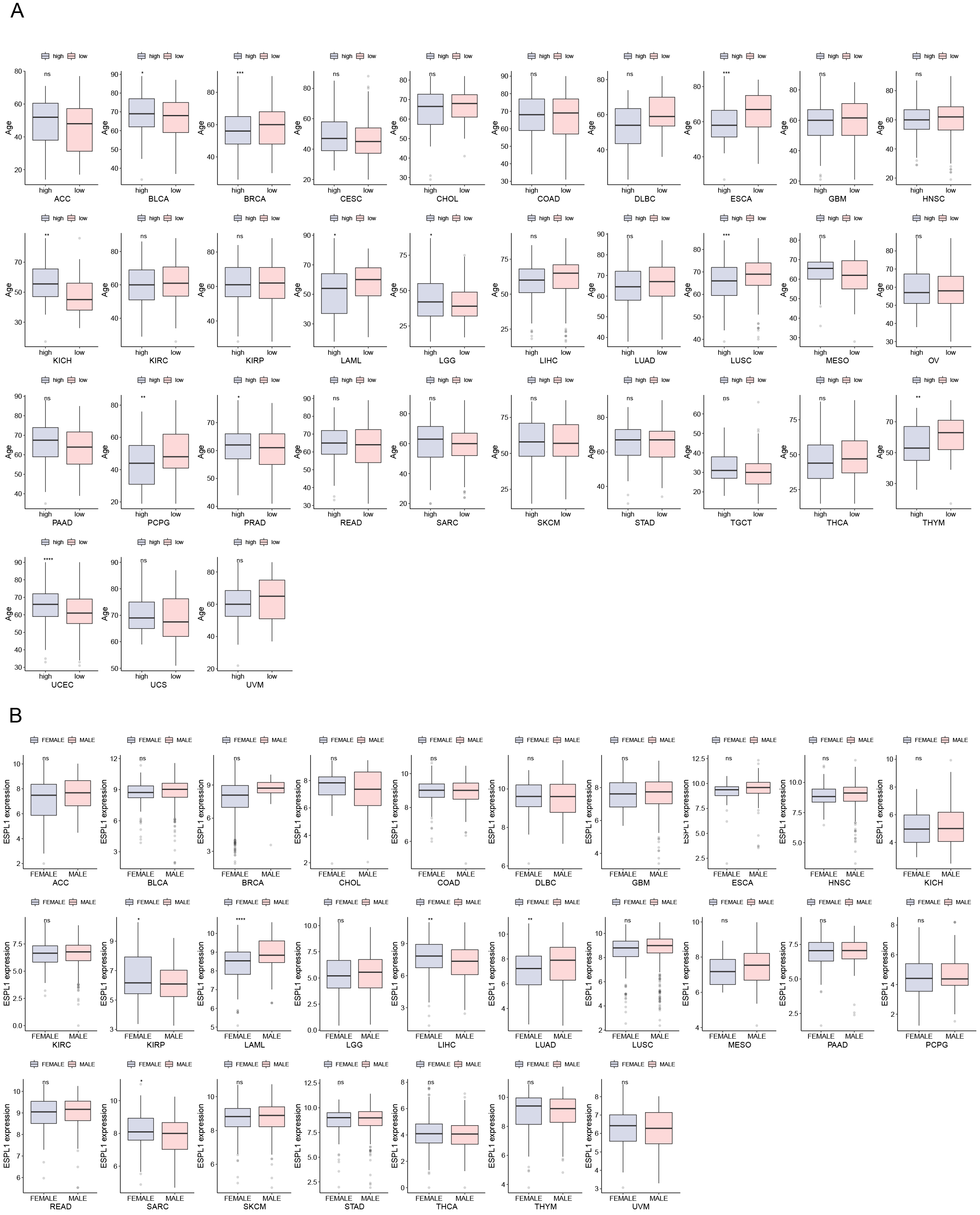

Supplement: Supplementary Figure 3 — Relationship between ESPL1 expression and clinical information. (A) Box plots showing the relationship between ESPL1 expression and age. (B) Box plots showing the relationship between ESPL1 expression and gender. [file Image_3.jpeg]

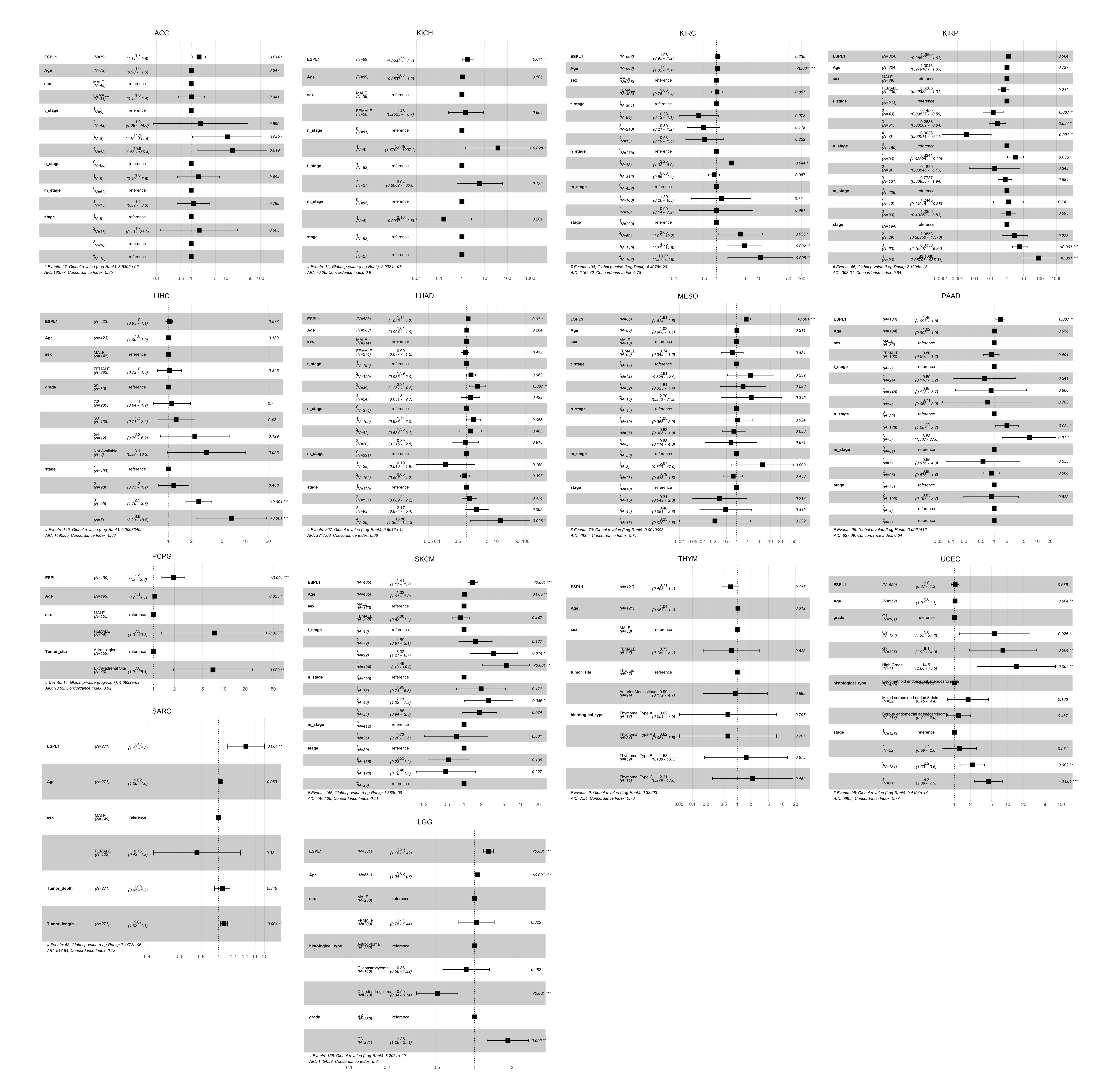

Supplement: Supplementary Figure 4 — Multivariate survival analysis based on ESPL1 expression and multiple clinical information. [file Image_4.jpeg]

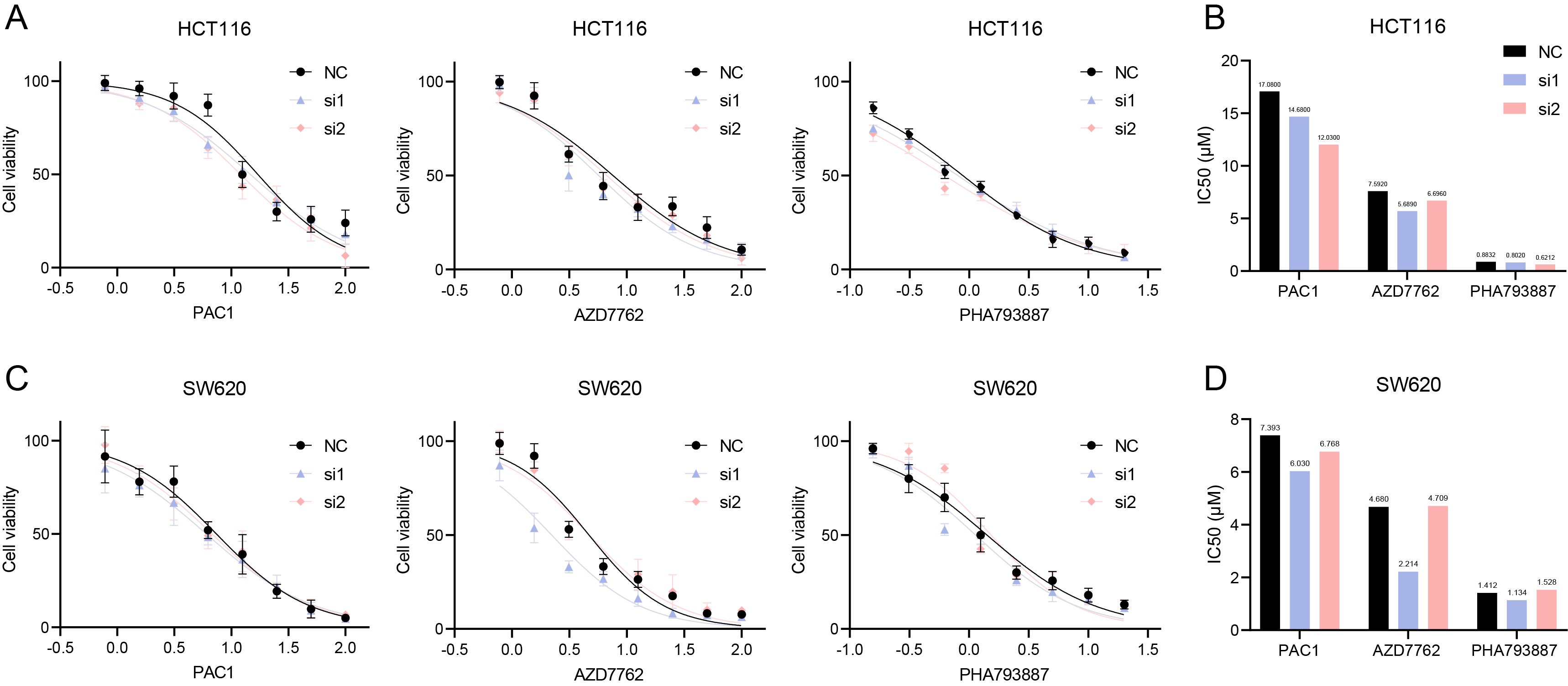

Supplement: Supplementary Figure 5 — The IC50 values of HCT116 and SW620 cells after siRNA-mediated interference of ESPL1 expression. (A and C) IC50 curves of three drugs in HCT116 and SW620 cells. (B and D) Column chart comparing IC50 values. [file Image_5.jpeg]
